# Perception of nephrology in Europe: a strategy to improve recruitment of motivated fellows

**DOI:** 10.1093/ckj/sfae326

**Published:** 2024-11-30

**Authors:** Talia Weinstein, Nadine Vogelsang, Sandor Sonkodi, Itzchak Slotki, Beatriz Martín-Carro, David Lappin, Jorge B Cannata-Andía, the UEMS Renal Section and Board, Uyen Huyn-Do, Aikaterini Papagianni, Theofanis Apostolou, Michael Ott, Peter J Heering, Andreja Marn-Pernat, Vera Certikova, Mikko Haapio, Bo Broberg, Halima Resic, Mai Rosenberg, Mustafa Arici, Anibal Ferreira

**Affiliations:** Department of Nephrology, Tel Aviv Medical Center, Tel Aviv University Medical School, Tel Aviv, Israel; Universitätsklinikum Münster, Medizinische Klinik D, Münster, Germany; First Department of Medicine, Faculty of Medicine, Szeged University, Szeged, Hungary; Adult Nephrology Unit, Shaare Zedek Medical Center, Faculty of Medicine, Hebrew University of Jerusalem, Jerusalem, Israel; Instituto de Investigación Biosanitaria del Principado de Asturias (ISPA), Oviedo, Spain; Department of Nephrology, Galway University Hospital and National University of Ireland, Galway, Ireland; Department of Medicine, Universidad de Oviedo, Oviedo, Spain

**Keywords:** medical training, mentorship, motivated recruitment, nephrology career

## Abstract

**Background:**

The shortage of applications for fellowships in nephrology is a worldwide challenge. This is the first survey to explore in Europe the reasons physicians choose (and do not choose) a career in nephrology.

**Methods:**

An anonymous questionnaire was sent to the presidents of societies that are members of the European Renal Association (ERA), who invited trainees and nephrologists to respond. Statistical analysis was performed using SPSS v.26. (SPSS Inc., Chicago, IL, USA). Continuous variables were compared by Student's *t*-test or by one-way ANOVA.

**Results:**

Responders included 516 (49%) females and 542 (51%) males. They comprised 278 (26%) trainees, and 780 (74%) nephrologists. The majority (64%) believe that students have an unfavourable perception of nephrology. For trainees, nephrology is not considered an attractive option due to ‘chronically ill patients’ (35%), ‘lack of contact during undergraduate training’ (37%), ‘nephrology is too challenging’ (38%), ‘poor remuneration’ (22%), ‘negative role models’ (15%), and ‘long working hours’ (14%). The factors with the greatest impact on choosing a career include a positive role model (46%), practical experience during medical school and early postgraduate training (42%).

**Conclusion:**

Trainees emphasize that work–life balance is very important for the younger generation. A strong mentorship along with early engagement is associated with a higher likelihood of pursuing a career in nephrology. It is crucial to create a strategy that will provide a positive experience, renew the interest in nephrology careers and ensure enough nephrologists to treat the growing number of patients with kidney disease.

KEY LEARNING POINTS **What was known:**

- The pursuit of a nephrology career is progressively declining, and as a result there is a growing shortage of nephrologists.
- The decreased number of nephrologists may not sustain in the near future the demands carried by the increasing number of patients with chronic kidney disease.
- Several recent US surveys have found that social, economic, working, and educational aspects are responsible for this worrying scenario.
- In Europe, the overall perception of the declining interest in a nephrology career is similar to the US findings, however, there are no European data addressing this important issue.

**This study adds:**

- The present European survey has identified several factors associated with the decreased interest in a nephrology career in Europe.
- The most prominent negative causes include poor exposure to nephrology in medical school, a negative work–life balance with long inflexible working hours, a challenging and difficult medical speciality with a proportionally poor remuneration.
- The positive influences include good practical experience during postgraduate training and medical school, an encouraging mentor role model, and the opportunity to participate in research.

**Potential impact:**

- The teaching of nephrology in medical schools should always be performed by experienced and dedicated nephrologists able to generate enthusiasm and passion for the speciality.
- There is a need for early exposure to renal pathophysiology and renal medicine in the medical school curriculum and it is advisable to include compulsory rotations in nephrology in the medical training.
- It is necessary to nurture the mentoring/role models in medical schools and during the medical training of nephrology.
- Adjust the working hours and shifts to obtain a better work–life balance.

## INTRODUCTION

In recent years, the pursuit of a nephrology career is declining progressively, thus aggravating the situation of a growing shortage of nephrologists worldwide. The decreased number of trainees may not sustain the workforce demands posed by the increasing numbers of patients suffering from kidney disease [1].

The 2019 Global Kidney Health Atlas reported that >70% of countries have a shortage of nephrologists [2], confirming previous reports [3–6]. In the 2022 US Nephrology Match, only 69% of fellowship positions and 52% of tracks were filled [7]. Recently, it has become a growing concern in European countries as well. In contrast to countries with a national allocation programme for medical training positions, in Europe there is a lack of data, mainly due to the heterogeneous training systems.

To elucidate the causes for the declining interest in nephrology, several surveys were conducted, mainly in the USA, over the last decade. They addressed various populations including medical students, internal medicine, and nephrology fellows as well as staff nephrologists involved in teaching programmes [8–13]. These studies revealed several motives that discourage the young physicians from deciding to become a nephrologist. The main reasons include unstimulating renal pathophysiology courses in medical school; disheartening inpatient experiences; lack of mentorship, lack of advances in the field of CKD, low monetary compensation, and perceived workload of nephrologists as one that is demanding and offers little reward.

To evaluate the factors that play a role in the fading interest in choosing a nephrology career in European countries, we conducted a pan-European survey addressed to trainees and certified nephrologists with the goal of identifying perceptions, attitudes, and barriers that influence this decision. We sought to provide insight into how to increase interest in this

speciality and to assist in recruiting the next generation of nephrologists.

## MATERIALS AND METHODS

The European Union of Medical Specialists (UEMS) Renal Section and Board designed an anonymous questionnaire with 268 questions (45 main questions plus items), which consisted of dichotomous (yes/no/not know) and multiple-choice as well as open-ended questions, which permitted free text responses.

Starting in January 2017, and throughout the whole year, three waves of questionnaires (with the same content), were sent to the European nephrology societies that are members of the European Renal Association (ERA). The National ERA presidents invited all nephrology trainees and certified nephrologists to respond. The data obtained from the collected answers were analysed first cumulatively, then stratified by gender, country, and status (trainee, trainer, nephrologist not involved in training).

### Data analysis

Statistical analysis was performed using SPSS v.26. (SPSS Inc., Chicago, IL, USA). Continuous variables were compared by Student's *t*-test or by one-way ANOVA. Frequencies without grouping were compared by chi-squared test for goodness of fit (fit to uniform distribution). The frequency of the distribution of variables between groups was compared using the chi-square test. Pairwise comparisons of percentages were performed by *z*-test. All answers were analysed using R https://www.r-project.org/ The ‘tm’ package https://cran.r-project.org/web/packages/tm/tm.pdf was used to find the most frequent terms among groups, which was used to select the 28 questions that were used for the present analysis (Supplementary Table).

## RESULTS

A total of 1058 nephrologists from 28 countries answered the questionnaires. We calculated the percentage of responders according to the number of nephrologists as listed in the ERA registry, having a heterogeneous rate of responses (>25%) three countries; (10–24.9%) 10 countries; (5–9.9%) five countries; and (<5%) nine countries. Due to the heterogeneity and very small number of respondents from some countries, we did not compare all responses by country. Nevertheless, as the number responses were enough to perform several analyses (*n* = 1058), we decided to group the total of responders according to their number: good, >50 responders (seven countries); medium, between 49 and 20 responders (eight countries); and poor, <20 responders (six countries). This categorization allowed us to investigate, using randomly five important questions selected from the questions using the ‘tm package’, any bias or differences in the type of response (Yes) between the good, medium, and poor responders. The questions in which there were >20% of ‘not known’ answers (15 cases), were not considered in the comparative analysis.

Table 1 shows the comparison of the mean percentages and ranges of the ‘yes’ responses in five important questions of the survey, (good, medium, and poor responders). As can be appreciated, independently of the country, there were no relevant differences in the percentages of answers in the three categories of responders.

**Table 1:** Percentages (mean and range) of positive answers (Yes) in countries with >50 responses (good responders, seven countries), 20 to 49 responses (median responders, eight countries), and <20 responders (poor responders, six countries).

		>50 responses	20–49 responses	1–19 responses
Total number of responses: Yes	878	569	240	69
Mean rate of yes responses	Mean (%)	Range (%)	Range (%)	Range (%)
Do you think there are currently good job opportunities for nephrologist in your country?	48	26–69	21–86	10–92
Some surveys describe a lack of role models as a major reason for young physicians not to choose nephrology, do you think this is the case compared to competing specialties?	40	31–49	21–42	18–69
Most medical students have an unfavourable perception of nephrology and considers nephrology a less attractive training option?	64	31–93	28–69	60–84
Do you think this negative perception changes after a nephrology rotation in most students?	78	70–88	62–89	60–100
Would you choose nephrology as a career again?	76	61–90	68–94	63–90

Total number or responses 1058. The responses with more than 20% of ‘I do not know’ were not included in the analyses (15 answers).

Countries with more than 50 answers (Greece, Italy, Spain, Portugal, Hungary, Denmark, Switzerland), between 20 and 49 answers (Austria, Croatia, France, United Kingdom, Norway, Finland, Ireland, Czech Republic), between 1 and 19 answers (Slovenia, Sweden, Israel, Poland, Germany).

The responders included 516 (49%) females and 542 (51%) males. There was a significant difference in the age of females compared to males (40.5 ± 10.4 vs. 47.1 ± 12.9; *P *< .01). The distribution of the professional status was as following: 278 (26%) trainees and 780 (74%) certified nephrologists of whom 387 (49%) were not involved in a medical training programme, but their opinion is very important as non-academic centres provide employment prospects for a great number of trainees. Among those involved in a training programme, there were significantly more trainer males than females (62.3% vs. 37.7%; *P *< .001), whereas among the trainees there were significantly more females (62.4% vs. 37.6%; *P *< .001). There was no gender difference in the group of nephrologists not involved in training.

In all categories studied, there was no significant difference between the responses of females and males regarding factors influencing nephrology as a career speciality. Sixty-six percent of respondents work in a public hospital affiliated with a university, 23% work in a non-affiliated public hospital, 8% work in a private dialysis unit, and 3% work in a private hospital, of which half are associated with a university. Table 2 summarizes the main reasons leading to the shortage of nephrologists as reported in recent US surveys performed with cohorts of at least 600 participants [10–19].

**Table 2:** Main reasons for the shortage of nephrologists mentioned in recent US surveys performed with more than 600 participants (references 10–19).

Reasons
Long work hours with a poor work–life balance, challenging work schedules
Low economical compensation
Chronic and difficult patients with low opportunity for new procedures
Shortage of role model mentors
Lack or poor previous exposure to nephrological patients in the medical school and in the training
Teaching of nephrology in the hands of no nephrologists in a greater percentage of medical schools (up to 50% of cases)
Deficient teaching of renal physiology and the renal medicine

**Figure 1:**
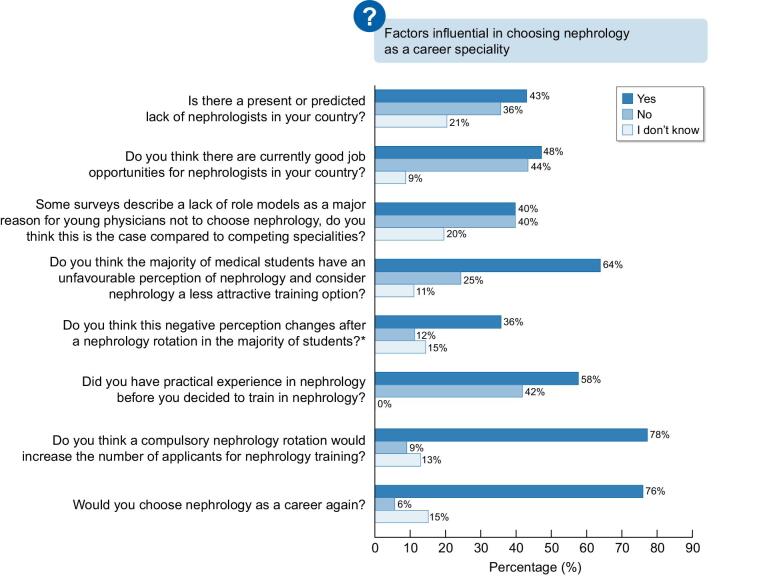
The status of nephrology in European countries and the factors influencing the choice of nephrology as a career speciality.

Figure 1 describes the status of nephrology in Europe and the factors influencing the choice of nephrology as a career speciality. There is a significant difference between various European countries regarding a predicted lack of nephrologists (*P *< .001). Most respondents think that medical students have an unfavourable perception of nephrology (*P *< .0001) and think that a compulsory rotation in nephrology would increase the number of applicants for nephrology training (*P *< .0001).

**Figure 2:**
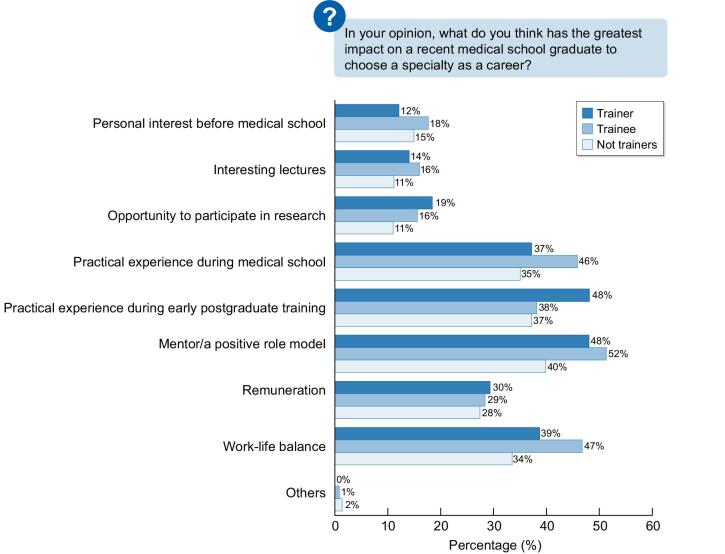
The elements that affect the decision of a recent medical graduate to choose a specific career.

Figure 2 describes the elements that affect the decision of a recent medical graduate to choose a specific career. For trainees, the most important factor is a mentor or a positive role model. In their viewpoint, life-work balance is significantly more important than remuneration.

**Figure 3:**
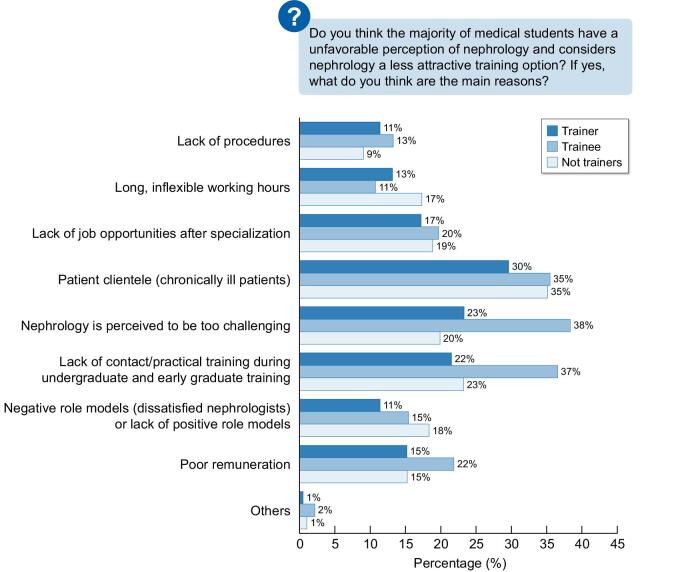
The opinions on the reasons why medical graduates have an unfavourable opinion on choosing nephrology.

Figure 3 describes the opinions on the reasons medical graduates have an unfavourable opinion on choosing nephrology. The significant differences between trainees and nephrologists are that trainees perceive nephrology to be too challenging (*P *< .0001), and that there is a lack of practical training during early graduate periods. In addition, the chronically ill patients deter the trainees.

**Figure 4:**
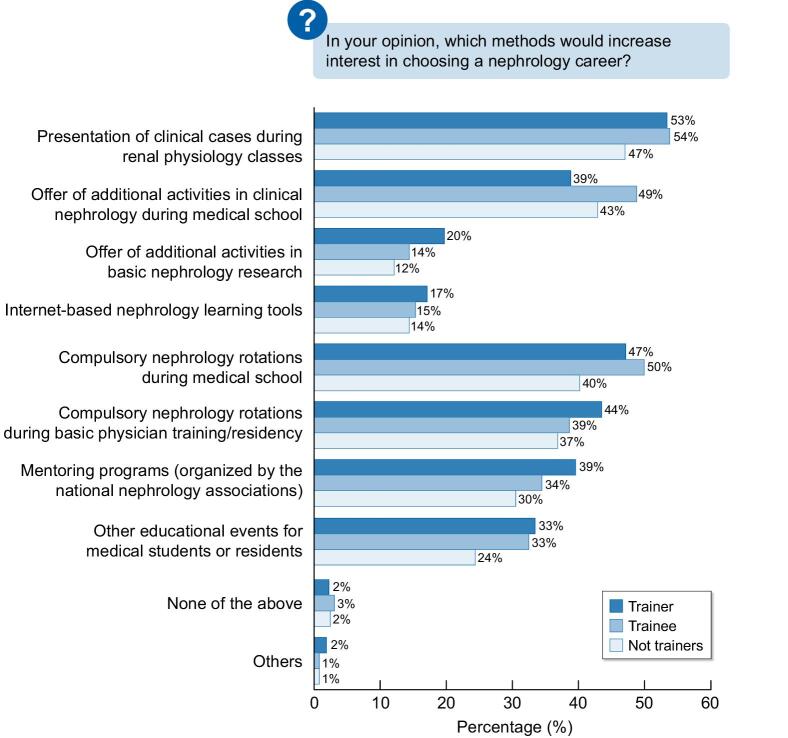
The perceived methods that would encourage more medical school graduates to choose nephrology as a career.

Figure 4 describes the perceived methods that would encourage more medical school graduates to choose nephrology as a career. The main factors are presentation of clinical cases during physiology classes, compulsory nephrology rotation during medical school and residency, and additional nephrology activities during medical school.

The final question of the survey was ‘Would you choose nephrology as a career again?’ Despite the described weaknesses of the profession, a surprising 76% of respondents answered that they would (*P *< .0001). A further analysis of the reasons for re-choosing a nephrology career found significant positive correlations with the following factors: interesting lectures (*P *< .02); practical experience during early postgraduate training (*P *< .0001); and a mentor/positive role model (*P *< .002).

## DISCUSSION

In recent decades, there has been a worldwide declining interest in choosing nephrology as a medical speciality, while the prevalence of CKD has been increasing [2]. This decline exists at academic medical centres as well as at private nephrology practices. As the training is heterogeneous in Europe, the present study explored the different viewpoints of nephrologists in 28 European countries. This is the first large-scale survey in Europe to investigate the reasons physicians choose (or do not choose) a career in nephrology. Several factors emerged as responsible for the current scenario; some are related to changes in the professional expectations of the younger generation that have evolved in the last decade, and others are mainly related to factors associated with the deficiencies, errors, defects, and weakness of the pre- and postgraduate education in nephrology.

Nephrology education takes place mainly in public hospitals, but there are important variations in the duration and assessment of training among the European countries. While the development of a standard, pan-European nephrology-training programme is not realistic, the European Union of Medical Specialists (UEMS) Renal section believes that this does not diminish the need for improving coordination of training [14]. The UEMS Renal Section and the ERA have been working jointly to harmonize European nephrology training and to establish the European Certificate in Nephrology [15]. In line with this initiative, the UEMS Renal Section embarked on the project of surveying the opinions on choosing a nephrology career.

To the best of our knowledge, this is the first survey to address the nephrology communities within the ERA. We studied subgroups of physicians working in nephrology: trainees, nephrologists involved in a training programme, and nephrologists not involved in training. It was of interest to investigate these three groups of nephrologists to evaluate differences in their opinions. We assessed attitudes regarding the following factors: personal interest, job opportunities, remuneration, education in medical school and residency training programmes, access to mentors, complicated patient populations, and early exposure to nephrology, work–life balance, research opportunities, and procedures.

The results demonstrate that 64% of respondents believe most medical students have an unfavourable perception of nephrology and consider nephrology a less attractive training option. Both trainees and certified nephrologists share this opinion, although it is more prevalent in the trainee group. The trainees identify the most influential causes as including ‘medically complicated and chronically ill patients’ (35%), ‘lack of contact during undergraduate and early graduate training’ (37%), and ‘nephrology is too challenging a speciality’ (38%). Other factors are ‘lack of job opportunities after specialization’ (20%), ‘poor remuneration’ (22%), ‘negative role models’ (dissatisfied nephrologists) (15%), and ‘long inflexible working hours’ (14%).

As for the evaluation of factors with the greatest impact on choosing a career on medical school graduation, all respondents believe they include a mentor and/or positive role model (46%), practical experience during medical school (39%), and practical experience during early postgraduate training (42%). Practical experience during medical school is significantly more important in the trainee group, compared to the others. Trainees stated that the most important factors for their own decision-making were a mentor/positive role model (43%), practical experience during postgraduate training (41%) and medical school (28%), work–life balance (25%), and opportunity to participate in research (15%).

The reasons for not choosing nephrology are multifactorial and share common characteristics between countries. In most countries, practical experience in nephrology is not compulsory during medical education (43%), and in only half of the medical schools, nephrologists teach the courses in nephrology. Therefore, there are fewer opportunities to mentor students. In all studied groups, a factor of major importance in the decision to choose a nephrology career is the presence of mentors and role models during medical school and internal medicine training. This is significantly more important for trainees. Seventy-eight percent of the respondents think that compulsory nephrology rotation would increase the number of applicants for nephrology training.

An additional element of our survey was the exploration of specific measures that could increase the interest in nephrology. The responses were similar in all subgroups: early exposure in medical school and during internal medicine residency is an important channel through which the profession may be introduced. Trainees believe that introducing clinical cases during physiology classes and performing additional clinical activities would enhance the selection of a nephrology specialization.

The findings in our survey have identified key elements that influence the choice of nephrology as a career in Europe. Although the grounds for the low attractiveness of a nephrology career vary among countries across the world, features such as our findings emerge from multiple surveys involving medical students, internal medicine residents, nephrology fellows, and nephrology programme directors.

Most of the surveys conducted during recent years involving large cohorts of medical students and internal medicine residents, mainly in the USA, have systematically shown similar results to this European study concerning the reasons that make nephrology less attractive as a medical career (Table 2) [10–19].

Many of the reasons are related to social and professional priorities of the new generation of medical students. There is no doubt that a complicated work–life balance, mainly due to demanding shifts and the tiring care of chronically ill patients are in the core of the nephrology practice. These drawbacks have been present since the establishment of the nephrology speciality and have always been a challenge for the dedicated nephrologist. In recent decades, we have also witnessed decreased remuneration of the nephrologists compared to other less demanding medical specialties, thus decreasing the motivation to choose it as a career. An additional aspect that has emerged is the notion that the complexity of nephrology practice is very challenging and deters young physicians.

Considering these observations, the goal of the UEMS Renal Section and Board in designing the present survey was to identify the topics that should be addressed in planning a novel approach to attract young physicians to nephrology.

The present study indicates that apart from the work–life balance issues, a change is required regarding nephrology education in medical school and during the training programmes. It was very surprising to discover that in only 50% of European medical schools nephrology is taught by nephrologists, and that nephrology rotation is not mandatory in many programmes. It is obvious that a lack of exposure to the wide scope of our profession prevents the possibility of engaging young doctors to choose our speciality. Indeed, the responses of trainers, trainees and certified nephrologists supported the opinion that nephrology rotation should be compulsory. The results depicted in Figs1 to 4 clearly show there are many actions, methods, and tools that can be implemented to improve the perception and attraction for a nephrology career. One of the most important factors that emerges from the survey is the importance of a positive dedicated mentorship for the young trainee.

Despite the negative viewpoints that emerge from the survey, most respondents state that they would choose nephrology as a career again. This demonstrates that despite the previously mentioned drawbacks, when renal medicine is well taught and practised it becomes an attractive and respected speciality. Therefore, it is our duty to implement novel teaching methods and improve mentorship programmes to renew the interest in practising nephrology among the students and young physicians. It has been demonstrated that supervising faculty involvement and behaviours play a central role in shaping attitudes. It has been suggested that our own professional dissatisfaction with the speciality and burnout is transparent to medical students and residents who are deciding on career choices and probably act as a deterrent to choosing nephrology [20, 21].

In summary, to obtain a highly trained nephrologist, the curriculum should engage students at an early stage, captivate their interest, develop their skills, and be delivered by educators who are passionate about teaching and are role models in the field [4]. In ours and in previous surveys, students report minimal exposure to nephrology during their clinical rotations. Embracing interventional nephrology and formally incorporating it into our training programmes may help our speciality attract more highly qualified trainees [22].

To identify the practices of institutions successfully generating nephrology career interest, the American Society of Nephrology Workforce Committee developed the ‘Best Practices Project’ to isolate translatable methods from the top nephrologist-producing institutions. Four major themes contributed to these schools’ successes: (i) nephrology faculty interaction with medical students; (ii) clinical exposure to nephrology and clinical relevance of renal pathophysiology materials; (iii) use of novel educational modalities; and (iv) early exposure to the breadth of nephrology practice [23]. A very recent perspective reinforced the importance of engaging trainees by enriching early elective experiences to secure the future of the nephrology workforce [24]. It has been suggested that a positive perspective for nephrology be presented to the next generation, offering a medical discipline that is both complex and beautiful at the same time [25].

To the best of our knowledge, this is the first survey performed in 28 European countries on this topic. The strength of this study is the inclusion of many respondents comprising trainers, trainees, and certified nephrologists from non-training centres and the comparison between their opinions. A limitation of our study is the fact that only 279 trainees answered the survey, and their voice is of utmost importance. However, 395 respondents to the survey are nephrologists involved in academic training, most of them were young, and their answers together with the voice of the 387 non-academic certified nephrologists provide useful information on how teaching is actually performed. Owing to the very small number of respondents from some countries, we did not compare all responses by country, yet we performed an analysis to enquire whether the responses of the different countries were equivalent (Table 1). The results show that the replies of the good, medium, and poor responding countries were comparable and followed a similar trend.

On the basis of the conclusions of our survey, we propose several actions to attract the next generation of nephrologists. These include: (i) increased involvement of nephrologists in the medical school curriculum by providing early exposure to nephrology and teaching the clinical applicability of renal physiology; (ii) promotion of mentoring/role models in medical schools and internal medicine training to nurture the interest in nephrology; and (iii) improved involvement with nephrology trainees by recognizing frustration, encouraging a suitable work–life balance, and reducing burnout. We believe that even on implementation of all the previously mentioned measures, the most powerful incentive for the young physician to choose a nephrology career is a dedicated nephrology mentor who conveys passion and fulfilment.

## Supplementary Material

sfae326_Supplemental_File

## Data Availability

The data underlying this article will be shared on reasonable request to the corresponding author.

## APPENDIX

Members of the Union Européenne des Médecins Specialistes (UEMS, European Union of Medical Specialists) Renal Section and Board who collaborated in the publication:

Uyen Huyn-Do (Switzerland), Aikaterini Papagianni (Greece), Theofanis Apostolou (Greece), Michael Ott (Sweden), Peter J. Heering (Germany), Andreja Marn-Pernat (Slovenia), Vera Certikova (Czech Republic), Mikko Haapio (Finland), Bo Broberg (Denmark), Halima Resic (Bosnia-Herzegovina), Mai Rosenberg (Estonia), Mustafa Arici (Turkey), and Anibal Ferreira (Portugal).

The UEMS Renal Section acknowledges with sorrow the recent passing of Professor Sankodi.
