# Supplementary material for: Perception of nephrology in Europe: a strategy to improve recruitment of motivated fellows
Source: Clin Kidney J. 2024 Nov 30;17(12):sfae326. doi: 10.1093/ckj/sfae326 (PMC11833319; doi:10.1093/ckj/sfae326)
Supplement: sfae326_Supplemental_File [file sfae326_Supplemental_File.docx]

**Supplementary table.** Questions (and items) selected using the “tm package”.

| Nº | Question |
| --- | --- |
| 1 | How old are you? |
| 2 | Are you male or female? |
| 3 | Are you currently involved in a medical training program? |
| 4 | At which kind of facility do you work? |
| 5 | At which kind of institution is your program located? |
| 6 | In which country are you working or training? |
| 7 | What is your current level of professional training? |
| 8 | Is there a present or predicted lack of nephrologists in your country? |
| 9 | Do you think there are currently good job opportunities for nephrologists in your country? |
| 10 | In many surveys from non-European countries, the poor remuneration in nephrology compared to other internal medical subspecialties is described, is that the case in your country? |
| 11 | In your opinion, has there been a change in interest in nephrology training in the recent years? |
| 12 | In your opinion, what do you think has the greatest impact on a recent medical school graduate to choose a specialty as a career? |
|  | Personal interest before medical school |
|  | Interesting lectures |
|  | Opportunity to participate in research |
|  | Practical experience during medical school |
|  | Practical experience during early postgraduate training (e.g. residency/basic physician training) |
|  | Mentor/ a positive role model |
|  | Remuneration |
|  | Work-life balance |
|  | Other |
| 13 | Which WAS the most important factor for you? |
|  | Personal interest before medical school3 |
|  | Interesting lectures4 |
|  | Opportunity to participate in research5 |
|  | Practical experience during medical school6 |
|  | Practical experience during early postgraduate training (e.g. residency/basic physician training)7 |
|  | Mentor/ a positive role model8 |
|  | Work-life balance9 |
|  | Other |
| 14 | Some surveys describe a lack of role models as a major reason for young physicians not to choose nephrology, do you think this is the case compared to competing specialties? |
| 15 | Do you think the majority of medical students has an unfavorable perception of nephrology and considers nephrology a less attractive training option? |
| 16 | If yes, what do you think are the main reasons? |
|  | Lack of procedures |
|  | Long, inflexible working hours |
|  | Lack of job opportunities after specialization |
|  | Patient clientele (chronically ill patients) |
|  | Nephrology is perceived to be too challenging |
|  | Lack of contact/practical training during undergraduate and early graduate training |
|  | Negative role models (dissatisfied nephrologists)/lack of positive role models |
|  | Poor remuneration |
|  | Other |
| 17 | Do you think this negative perception changes after a nephrology rotation in the majority of students? |
| 19 | Is practical experience in nephrology compulsory during medical education in your country/department? |
| 20 | Did you have practical experience in nephrology before you decided to train in nephrology? |
| 21 | Do you think a compulsory nephrology rotation would increase the number of applicants for nephrology training? |
| 22 | Do you think it makes a difference if the first nephrology related class (usually renal physiology) is taught by a clinical nephrologist or a physiologist? |
| 23 | Who teaches this class at your institution? |
|  | Nephrologist |
|  | Internal medicine specialist |
|  | Physician of other specialty (not internal medicine) |
|  | Life scientist (biologist, physiologist) |
|  | I don't know |
| 24 | When students or residents do a nephrology rotation in your department, in which setting does the training take place? |
| 25 | Which topics are the students/residents mainly exposed to? |
|  | CKD |
|  | Acute kidney injury |
|  | Electrolyte/acid-base disorder |
|  | Dialysis |
|  | Intensive Care Nephrology |
|  | Transplantation |
|  | Hypertension |
|  | Glomerulonephritis/immunological diseases |
|  | Apheresis |
|  | The students/residents rotate through all of the above in a scheduled manner |
| 26 | In your opinion, which methods would increase interest in choosing a nephrology career? |
|  | Presentation of clinical cases during renal physiology classes |
|  | Offer of additional activities in clinical nephrology during medical school (e.g. shadowing of a consultant, attending nephropathology sessions, participating in clinical nephrology studies) |
|  | Offer of additional activities in basic nephrology research |
|  | Internet-based nephrology learning tools |
|  | Compulsory nephrology rotations during medical school |
|  | Compulsory nephrology rotations during basic physician training/ residency |
|  | Mentoring programs (organized by the national nephrology associations) |
|  | Other educational events for medical students or residents organized by the national nephrology associations (e.g. special courses at national congresses/ reduced entrance fees for students and residents) |
|  | None of the above |
| 27 | Would you choose nephrology as a career again? |
| 28 | Do you have any other suggestions on how to raise interest in nephrology training? (free answer) |
